# Lumbopelvic hyperlordosis is linked to higher femoral head coverage, lower femoral anteversion and younger age at periacetabular osteotomy

**DOI:** 10.1002/ksa.12587

**Published:** 2025-01-16

**Authors:** Maximilian Fischer, Lars Nonnenmacher, Andreas Nitsch, Matthias R. Mühler, Alexander Möller, Andre Hofer, Georgi I. Wassilew

**Affiliations:** ^1^ Center for Orthopaedics, Trauma Surgery and Rehabilitation Medicine University Medicine Greifswald Greifswald Germany; ^2^ Department of Radiology and Neuroradiology University Medicine Greifswald Greifswald Germany; ^3^ Department of Radiology University of Wisconsin Madison Wisconsin USA

**Keywords:** developmental dysplasia of the hip, lumbopelvic alignment, pelvic tilt, periacetabular osteotomy, PROM

## Abstract

**Purpose:**

The dynamic alignment of the lumbar spine, pelvis and femur is increasingly studied in hip preservation surgery. However, the interaction between lumbopelvic alignment, acetabular and femoral morphology and its influence on patients' preoperative symptom burden remains poorly understood. The aim of this study was to evaluate whether lumbopelvic malalignment affects osseous hip morphology and exacerbates preoperative patient‐reported joint functionality in patients undergoing periacetabular osteotomy (PAO).

**Methods:**

One hundred thirteen patients were prospectively enroled in this single‐centre study. Sagittal lumbopelvic radiographs were used to divide the patients in accordance with their lumbopelvic alignment (pelvic incidence [PI]–lumbar lordosis [LL] mismatch) into a balanced (PI–LL: 10° and 10°/*n* = 60) and unbalanced alignment (PI–LL: <10° and >10°/*n* = 53) group. Intergroup analyses were performed for acetabular and femoral morphology as well as various patient‐reported outcome measures (PROMs) scores (modified Harris‐Hip, Hip Osteoarthritis Outcome, International Hip Outcome tool‐12 and University of California Los Angeles activity scale).

**Results:**

Patients with concomitant unbalanced lumbopelvic alignment due to hyperlordosis showed higher femoral head coverage and lower femoral anteversion (lateral centre‐edge angle 20.2° vs. 15.8°, *p* = 0.012/anterior wall index 0.47 vs. 0.36, *p* = 0.001/acetabular inclination 10.2° vs. 13.6°, *p* = 0.008/Femoral anteversion 21.3° vs. 28.2°, *p* = 0.041). Furthermore, these patients were significantly younger at the time of PAO (28.7 vs. 32.4 years, *p* = 0.020), even when there were no intergroup differences in all analyzed PROMs.

**Conclusion:**

Concomitant lumbopelvic deformity affecting the hip joint morphology could aggravate clinical symptoms leading to earlier presentation in patients undergoing PAO. Thus, the lumbopelvic balance needs to be carefully evaluated in clinical decision‐making in PAO patients and future research should focus on long‐term outcomes of patients with concomitant unbalanced lumbopelvic alignment.

**Level of Evidence:**

Level III, prognostic study.

**Abbreviations**
AI: acetabular inclination
AR: acetabular retroversion
AWI: anterior wall index
BHD: borderline hip dysplasia
FV: femoral version
HD: hip dysplasia
HOOS: Hip Osteoarthritis Outcome Score
iHOT‐12: International Hip Outcome Tool‐12
LCEA: lateral centre‐edge angle
LL: lumbar lordosis
mHHS: modified Harris Hip Score
MRI: magnetic resonance imaging
PAO: periacetabular osteotomy
PI: pelvic incidence
PROMs: patient‐reported outcome measures
PT: pelvic tilt
PWI: posterior wall index
SD: standard deviation
SS: sacral slope
UCLA: University of California Los Angeles activity scale

## INTRODUCTION

Periacetabular osteotomy (PAO) has gained considerable importance in recent decades [6]. PAO allows a three‐dimensional correction of congenital hip deformities to optimize joint loading [17, 21, 26]. Several predictors of outcomes following PAO including patient individual, as well as radiographically evaluable joint factors, amongst others are already known [3, 5, 10, 34, 40].

While the diagnostic focus has primarily been on the acetabular and femoral anatomy, the significance of the lumbopelvic relation has largely been overlooked. Starting with the increased understanding of femoroacetabular dynamics during weightbearing, the considerations of hip biomechanics have extended to the lumbo–pelvic–femoral axis [16, 30].

Although various studies have convincingly demonstrated the importance of the lumbopelvic alignment along with acetabular cup and femoral stem positioning in total hip arthroplasty, this aspect remains underrepresented in hip‐preserving surgery [27, 33].

However, it is increasingly becoming a focus of interest, as an optimal hip–spine interaction is crucial for a proper joint function [37]. Analyses of static radiographs have already demonstrated that pelvic tilt (PT) is associated with inadequate femoral head coverage in hip dysplasia (HD) as well as with a prominent crossover sign in acetabular retroversion (AR) [11, 29, 32]. Furthermore, minimal changes in PT have significant effects on acetabular parameters, such as the lateral and anterior femoral head coverage, raising the question of whether this represents a functional endogenous adaption or an additional morphologic component of hip biomechanics [16, 42]. Additionally, diagnostic cohort studies have reported conflicting results on differences in lumbopelvic relationships across various joint morphologies, making this topic and its clinical relevance a continued subject of debate in hip‐preserving surgery [8, 18, 24, 39].

In this context, a recent study in patients with femoroacetabular impingement syndrome highlighted the clinical relevance of lumbopelvic balance by reporting an association between patient symptoms, restricted joint functionality, limited lumbar flexion and PT [9, 28].

Despite these findings, a significant gap in the current literature is the lack of comprehensive studies systematically investigating the dynamic interaction of the lumbo–pelvic–femoral axis in hip‐preserving surgery by PAO. Closing these knowledge gaps is critical to support the development of new therapeutic approaches, identify individual risk factors for poor postoperative outcomes, and minimize complications.

Thus, this study aimed (i) to investigate the impact of lumbopelvic malalignment on hip morphology and (ii) patient‐reported joint functionality in patients undergoing PAO. It was hypothesized that unbalanced lumbopelvic alignment, including hyperlordotic and flatback deformities, influences osseous acetabular and femoral morphology, as well as patients' preoperative symptom burden.

## METHODS

### Study design

A prospective, review board‐approved, diagnostic cohort study evaluating 113 patients (114 hips, 76% female) with symptomatic HD (*n* = 58), borderline hip dysplasia (BHD) (*n* = 41) and AR (*n* = 15) was conducted in one high‐volume institution (Table 1).

**Table 1:** Patient characteristics, acetabular morphology and femoral version.

		Unbalanced	
		Hyperlordotic	
	Balanced	Flatback	*p*
Age (SD)	**32.4 (7.9)**	**29.1 (7.6)**	**0.029**
		***28.7 (7.8)***	***0.020***
		*31.4 (5.7)*	*0,732*
BMI (SD)	23.7 (2.5)	23.4 (3.2)	0.723
		*23.0 (3.1)*	*0.361*
		*25.2 (2.5)*	*0.229*
HD (% from total)	**37 (64)**	**21 (36)**	
		16 *(28)*	
		*5 (12)*	
BHD (% from total)	19 (46)	22 (54)	
		20 *(49)*	
		*2 (5)*	
AR (% from total)	**4 (27)**	**11 (73)**	
		10 *(67)*	
		*1 (6)*	
LCEA, ° (SD)	**15.8 (9.3)**	**19.7 (7.5)**	**0.028**
		***20.2 (7.4)***	***0.012***
		*16.4 (6.8)*	*0.974*
AI, ° (SD)	**13.6 (7.2)**	**10.9 (6.9)**	**0.037**
		***10.2 (6.6)***	***0.008***
		*14.9 (7.4)*	*0.656*
AWI (SD)	**0.36 (0.11)**	**0.45 (0.18)**	**0.012**
		*0.47 (0.17)*	***0.001***
		*0.32 (0.14)*	*0.195*
PWI (SD)	0.85 (0.14)	0.81 (0.15)	0.355
		*0.81 (0.15)*	*0.353*
		*0.82 (0.18)*	*0.601*
FV Murphy (SD)	**28.2 (13.3)**	**21.6 (12.9)**	**0.047**
		*21.3 (13.1)*	***0.041***
		*24.5 (11.5)*	*0.709*

*Note*: Mean with standard deviation (SD). Bold—significant intergroup difference *p* < 0.05.

Abbreviations: AI, acetabular inclination; AR, acetabular retroversion; AWI, anterior wall index; BHD, borderline hip dysplasia; BMI, body mass index; FV, femoral version; HD, hip dysplasia; LCEA, lateral centre‐edge angle; PWI, posterior wall index.

All included patients underwent PAO between January 2024 and July 2024. The treatment decision was made based on a combination of patient‐reported symptoms (refractory hip pain lasting more than 6 months with failed conservative therapy), physical examination, and radiographic parameters. All patients gave written informed consent prior to inclusion. Ethics approval (BB099/20a) was obtained from the local independent ethics committee of the University Medicine Greifswald according to the World Medical Association Declaration of Helsinki.

### Radiographic assessment

Each patient underwent lateral view radiographs of the lumbar spine, pelvis, and femur. This included a consecutive series of lumbopelvic radiographs in standing, relaxed‐seated (hip flexion 90° and femurs parallel to the floor) and deep‐seated position (maximal forward‐leaning and femurs parallel to the floor). The field of view included the lumbar spine, pelvis and femoral heads. Regarding the enlarged field of view, the source‐to‐detector distance was elevated to 180 cm. Examinations were performed on a Philips DigitalDiagnost C90 (Koninklijke Philips N.V.). With respect to the young age of our patient population, an adapted low‐dose radiation protocol was introduced.

**Figure 1:**
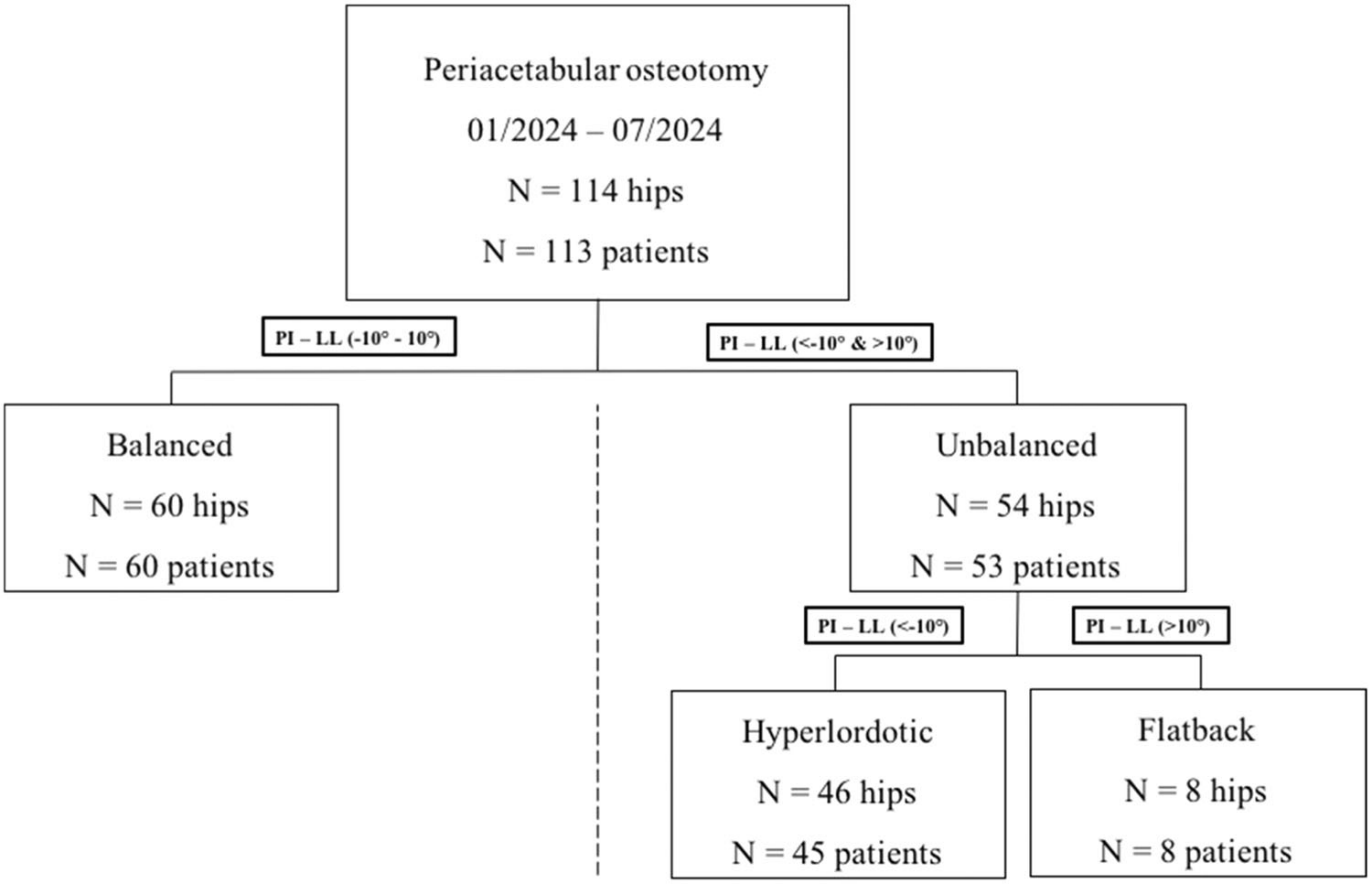
Flowchart of the study cohort. Lumbopelvic alignment was classified in accordance with their PI–LL mismatch on standing lumbopelvic radiographs. LL, lumbar lordosis; PI, pelvic incidence.

Radiographs were reviewed for pelvic incidence (PI), lumbar lordosis (LL), sacral slope (SS) and PT in all three positions mentioned before. Lumbopelvic alignment was classified as ‘balanced’ or ‘unbalanced’ including hyperlordotic and flatback deformity (Figure 1) using the PI–LL mismatch with a cut‐off of ±10° [31].

The lumbopelvic variables were measured as follows:

PI—Angle between a perpendicular axis to the midpoint of S1 endplate and the femoral head centre.

PT—Angle between the vertical axis and a line connecting the centre of the S1 endplate and the femoral head centre.

LL— Angle between L1 and S1 endplates.

SS—Angle between S1 endplate and a horizontal reference line.

Acetabular morphology was assessed by anteroposterior pelvic radiographs as well as axial and faux‐profile femoral views. HD was classified using a lateral centre‐edge angle (LCEA) cut‐off value of <18°, while BHD was defined through an LCEA between 18° and 25°. AR was defined with all three signs of AR (crossing‐over, posterior wall and sciatic spine sign) available on anteroposterior pelvic radiographs combined with an LCEA > 25° [25].

The femoral version was measured by magnetic resonance imaging (MRI) in accordance with the method described by Murphy [13]. The angle of femoral version (FV) was constructed by the femoral head centre, femoral neck base and condylar axis. MRI was performed in supine position using moving table technique in a 3 T full‐body scanner (Magnetom VIDA, Siemens). The ankles were fixed by a tourniquet to prevent sporadic rotation. Only slices in transversal orientation were acquired. The pelvis scan was extended to the distal edge of the lesser trochanter applying a T1w primarily introduced for muscle evaluation. The knees and the ankle joints were examined applying a T2 turbo spin echo sequence for fast acquisition.

### Data collection and statistical analysis

Preoperative PROMs were assessed by standardized questionnaire. The modified Harris Hip Score (mHHS) and the International Hip Outcome Tool‐12 (iHOT‐12) were used to determine hip joint functionality [4, 15]. Daily functionality and quality of life were assessed by the Hip Osteoarthritis Outcome Score (HOOS) using the subscales for symptoms, sport, pain, quality of life and activity in daily life with higher scores indicating higher functionality [41]. The University of California Los Angeles activity scale (UCLA) determined patient‐reported activity level [2].

Descriptive statistics were used to summarize the patient characteristics and outcomes. Data are reported as mean with standard deviation. Statistical analyses were performed using SPSS (version 29, IBM). Differences between the groups were tested for statistical significance by the Mann–Whitney *U* test for continuous variables. A *p* value less than 0.05 was considered statistically significant. A post hoc power analysis using G*power 3.1 was conducted to evaluate the primary research question regarding differences in acetabular morphology, utilizing the most commonly used parameter in literature (LCEA) and the most significant parameter in this study (AWI). Comparing lumbopelvic balanced and the primary research group (hyperlordotic deformity), the achieved test power (1 − *ß*) ranges between 0.75 (LCEA) and 0.95 (AWI).

## RESULTS

Overall, 53 patients (54 hips) presented with unbalanced lumbopelvic alignment, including hyperlordotic (46 hips) as well as flatback deformity (8 hips), and 60 patients (60 hips) presented with balanced alignment. Patients with concomitant hyperlordotic lumbopelvic deformity underwent PAO at a significantly younger age compared to patients with balanced alignment (28.7 vs. 32.4 years, *p* = 0.020). The mean BMI was 23.7 kg/m^2^ for unbalanced and 23.4 kg/m^2^ for balanced patients (*p* = 0.723) (Table 1).

**Figure 2:**
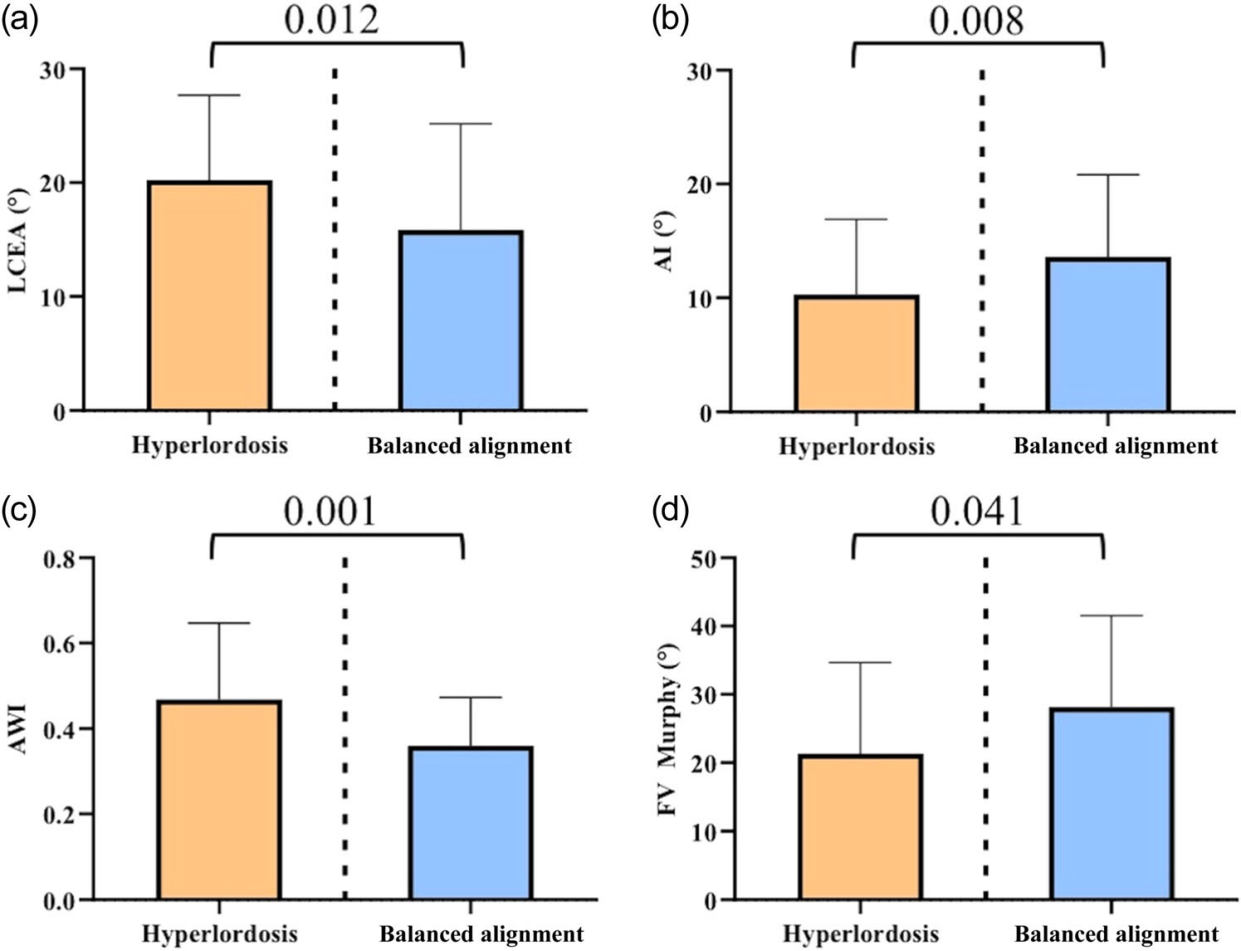
Balanced lumbopelvic alignment was associated with a dysplastic acetabular morphology and higher femoral anteversion. Intergroup difference of acetabular morphology, (a) lateral centre‐edge angle (LCEA), (b) acetabular inclination (AI), (c) anterior wall index (AWI) and (d) femoral version Murphy (FV). Intergroup difference—mean with standard deviation, *p* < 0.05.

Patients with balanced alignment had significantly lower lateral acetabular coverage compared to patients with concomitant lumbopelvic hyperlordosis (15.8° vs. 20.2°, *p* = 0.012) accompanied by a significant greater acetabular inclination (13.6° vs. 10.2°, *p* = 0.008) (Figure 2a,b). Additionally, the anterior femoral head coverage was significantly smaller in alignment‐balanced patients compared to patients with lumbopelvic hyperlordosis (0.36 vs. 0.47, *p* = 0.001) (Figure 2c). There was no significant difference in posterior femoral head coverage between the balanced and unbalanced group (0.85 vs. 0.81, *p *= 0.355).

MRI measurement of FV revealed significant differences between balanced and lumbopelvic unbalanced patients due to hyperlordotic deformities, with higher femoral version in patients with balanced lumbopelvic alignment (28.2° vs. 21.3°, *p* = 0.041) (Figure 2d).

Static lumbopelvic parameters showed significant differences between balanced alignment and concomitant hyperlordotic deformities in standing, relaxed‐seated and deep‐seated positions. The PI (57.2° vs. 46.5°, *p* < 0.001) and the standing PT (16.4° vs. 7.1°, *p* < 0.001) were significantly higher in lumbopelvic balanced patients compared to those with hyperlordotic deformities (Table 2). Furthermore, the analyses revealed a higher PT in the relaxed‐seated position (36.4° vs. 25.8°, *p* < 0.001) as well as deep‐seated position (11.9° vs. 5.1°, *p* = 0.001) in patients with balanced alignment compared to patients with concomitant lumbopelvic hyperlordosis (Table 2).

**Table 2:** Lumbopelvic parameters in standing, relaxed‐seated and deep‐seated position.

		Unbalanced	
		Hyperlordotic	
	Balanced	Flatback	*p*
Mean pelvic incidence, ° (SD)	**57.2 (11.2)**	**49.6 (12.6)**	**<0.001**
		***46.5 (8.2)***	< ***0.001***
		*66.6 (17.8)*	*0.087*
Mean lumbar lordosis—standing, ° (SD)	59.1 (10.9)	60.9 (11.1)	0.218
		*63.3 (8.1)*	*0.089*
		*48.0 (15.6)*	*0.056*
Mean sacral slope—standing, ° (SD)	41.3 (9.4)	39.7 (7.9)	0.449
		*39.6 (6.8)*	*0.252*
		*40.5 (12.3)*	*0.870*
Mean pelvic tilt—standing, ° (SD)	**16.4 (6.1)**	**8.9 (8.1)**	**<0.001**
		***7.1 (5.1)***	< ***0.001***
		*19.4 (12.5)*	*0.107*
Mean lumbar lordosis—seated, ° (SD)	29.8 (14.2)	32.7 (12.9)	0.504
		*33.8 (12.9)*	*0.381*
		*26.5 (11.1)*	*0.479*
Mean sacral slope—seated, ° (SD)	22.6 (9.8)	22.6 (9.2)	0.942
		*22.9 (9.3)*	*0.714*
		*20.6 (8.6)*	*0.497*
Mean pelvic tilt—seated, ° (SD)	**36.4 (10.7)**	**28.1 (11.5)**	**0.001**
		***25.8 (9.2)***	< ***0.001***
		*40.6 (14.8)*	*0.385*
Mean lumbar lordosis—deep seated, ° (SD)	−0.9 (10.2)	−1.9 (11.7)	0.805
		*−1.1 (11.1)*	*0.838*
		*−6 (13.9)*	*0.369*
Mean sacral slope—deep seated, ° (SD)	46.7 (12.1)	45.3 (10.8)	*0.284*
		*44.2 (10.0)*	*0.296*
		*51.0 (12.8)*	*0.558*
Mean pelvic tilt—deep seated, ° (SD)	**11.9 (11.7)**	**5.8 (12.2)**	**0.010**
		*5.1 (10.6)*	***0.001***
		*9.4 (18.4)*	*0.937*

*Note*: Mean with standard deviation (SD). Bold—significant intergroup difference compared to ‘Balanced’ *p* < 0.05.

The preoperative symptom burden was evaluated by joint functionality, activity, and quality of life scoring (Table 3). Especially, the quality of life (HOOS QoL) is negatively affected by the underlying hip pathology in both groups (25.6 − 26.7/100 points, *p* = 0.825). Comparing the preoperative PROMs between patients with balanced and unbalanced lumbopelvic alignment, there was no significant difference in all analyzed PROMs (Table 3).

**Table 3:** Preoperative patient‐reported outcome measures.

		Unbalanced	
		Hyperlordotic	
	Balanced	Flatback	*p*
mHHS (SD)	59.1 (16.5)	56.3 (15.9)	0.640
		*56.7 (16.2)*	0.836
		*53.8 (14.3)*	0.388
iHOT‐12 (SD)	44.7 (17.6)	46 (15.6)	0.545
		*46.7 (15.7)*	0.489
		*42.8 (14.7)*	0.876
UCLA (SD)	6.5 (2.4)	5.8 (2.7)	0.244
		*5.8 (2.7)*	0.238
		*5.7 (2.9)*	0.487
HOOS			
Pain (SD)	57.6 (17.4)	51.5 (20.3)	0.270
		*51.2 (19.7)*	0.298
		*48.8 (23.8)*	0.261
Sport (SD)	42.4 (25.6)	47.8 (22.4)	0.557
		*40.1 (25.8)*	0.401
		*45.8 (27.6)*	0.819
Symptom (SD)	59 (19.0)	52.6 (22.8)	0.318
		*52.8 (20.2)*	0.261
		*51.7 (31.9)*	0.547
Quality of life (SD)	26.7 (17.6)	25.2 (14.2)	0.825
		*24.8 (14.5)*	0.667
		*27.3 (12.3)*	0.891
Activity in daily life (SD)	71.7 (16.8)	64.0 (24.4)	0.427
		*64.8 (23.9)*	0.437
		*60.5 (26.1)*	0.351

*Note*: Mean with Standard deviation (SD). Intergroup difference compared to ‘Balanced’ *p* < 0.05.

Abbreviations: HOOS, Hip Osteoarthritis Outcome Score; iHOT‐12, International Hip Outcome Tool‐12; mHHS, modified Harris‐Hip score; UCLA, University of California Los Angeles activity score.

## DISCUSSION

The main findings of this diagnostic cohort study were (i) lumbopelvic hyperlordosis was associated with greater femoral head coverage and lower femoral anteversion in patients undergoing PAO and (ii) patients with concomitant hyperlordotic deformities underwent PAO at an early age even when they experienced no differences in preoperative patient‐reported hip functionality.

The current study found that HD and concomitant greater femoral version are more frequently observed in patients with balanced lumbopelvic alignment. In contrast, AR was associated with lumbopelvic malalignment. Thus, these results further increase the evidence of substantial differences in lumbopelvic alignment between instability and impingement‐driven hip deformities in PAO cohorts.

Primarily focused on the PT, it has already been shown that the radiographic acetabular appearance is sensitive to the lumbopelvic tilting and should be critically evaluated preoperatively [20, 22, 32]. Recently published data indicated that the biomechanical impact of PT is even more complex than affecting the radiographic acetabular appearance between the supine and standing position alone. It seems to be a relevant component of acetabular joint morphology as well [16, 39]. With a further improved understanding of the dynamic lumbo–pelvic axis, recommendations on cut‐off values for radiographic signs of impingement‐driven pathologic joint conditions were determined to improve the diagnostic accuracy while not observing the lumbo–pelvic–femoral axis comprehensively [38].

In this respect, the findings of the current study further extend the knowledge of hip biomechanics to the lumbo–pelvic–femoral axis in young, symptomatic patients undergoing PAO. The current data indicate that a restricted focus on PT in lumbopelvic assessment is insufficient, given the distinct interplay between lumbopelvic balance and spinal, acetabular and femoral parameters, which collectively form the lumbo–pelvic–femoral axis. Therefore, a comprehensive preoperative evaluation of the dynamic hip–spine interaction should include femoral and several lumbopelvic metrics, including PI, PT and LL, to optimize planning for hip‐preserving interventions.

Nevertheless, it remains a topic of major debate whether lumbopelvic alignment represents an endogenous adaption to counteract insufficient acetabular coverage, resulting in a higher mechanical contact stress with consecutive joint degeneration, or being a corresponding anatomical component of hip pathology [1, 12, 16, 19]. In on‐going studies, increased effort has been made to investigate changes in the lumbopelvic axis after PAO. Despite several methodological differences between the studies, and based on anteroposterior pelvic radiographs, minimal changes of the PT have been reported, indicating a compensatory mechanism [7, 14, 35]. In contrast, Haertleét et al. concluded in their study on 145 HD patients that the lumbopelvic alignment represents a component of pathology with differences even between HD phenotypes [16]. The result of the present study further emphasizes this point, showing that lumbopelvic hyperlordosis is present in both HD and AR. Interestingly, concomitant lumbopelvic hyperlordosis was even more frequently observed in AR patients and differences in PT remained evident across all analyzed positions of daily living. Thus, the lumbopelvic alignment represents a patient individual factor, which could be biomechanical compensation or even aggravate the hip pathology.

The constitutional character of the lumbo–pelvic–femoral axis in prearthritic hip diseases indicates the need for adaptions in current treatment strategies, and its clinical relevance requires further evaluation. In this context, the current study found that patients with hyperlordotic lumbopelvic alignment underwent PAO at a younger age compared to those with balanced lumbopelvic alignment. Even when the study did not find a greater preoperative symptom load in patients with hip deformity and concomitated unbalanced lumbopelvic alignment, the data indicate that concomitant lumbopelvic hyperlordosis could be a potential risk factor for earlier pain and decision for surgery in PAO patients. Thus, the current study extends the knowledge of aggravating pathologies as risk factors for early symptom presentation in hip preservation cohorts [36]. Furthermore, the present study results indicated that lumbopelvic malalignment is already present in cohorts undergoing hip preserving surgery and could potentially influence the timing of patients' decision to undergo surgery. In primary studies in THA cohorts, Kleeman‐Forsthuber et al. reported age‐related changes in lumbopelvic parameters over time in a largely older patient cohort [23]. Consequently, based on this literature and the current study results, the lumbopelvic relation should be considered even in young patient cohorts without extended degenerative hip joint diseases.

### Limitations

This study has several limitations that must be considered and discussed. First, the study included only patients receiving PAO to treat mild to severe HD and AR, and there could be an increased risk of selection and treatment bias. Thus, the results of our cohort study are not generable to all hip‐preserving indications. Additionally, the radiographic assessment mainly focused on osseous pathologies and accompanying soft tissue pathologies affecting the PROMs were not evaluated in this study. Future studies could extend the MRI diagnostics to 3D acetabular coverage and soft tissue evaluations. Next, hyperlordotic and flatback deformities were not equally distributed within the current study cohort, reflecting the fact of a prospective pilot study. Due to the rare condition of flatback deformity in this cohort, the study was underpowered by detecting small to moderate differences between the lumbopelvic balanced and flatback deformity group. Thus, these results could be the starting point for on‐going research on flatback deformity in hip preservation patient cohorts with a larger sample size.

### Future directions

The current study is the first to report PROMs in a PAO patient cohort facing the dynamic lumbo–pelvic–femoral interaction. While the present study focused on the preoperative influence of lumbopelvic malalignment on patients' symptom load and joint deformity, the question about its influence on postoperative long‐term outcomes remained unanswered. To address this question, prospective longitudinal studies on PAO outcomes in patients with concomitant hyperlordotic and flatback deformities are needed. Including the methodical strengths of the current study, such as assessing the sagittal lumbopelvic alignment and measuring FV using MRI with a standardized protocol, could further enhance the understanding of the lumbo–pelvic–femoral axis in PAO.

## CONCLUSION

In summary, concomitant lumbopelvic malalignment represents a relevant patient factor that impacts the acetabular and femoral hip morphology and serves as a risk factor for early symptom presentation in patients undergoing PAO. Future research on postoperative PROMs is warranted to illuminate the long‐term effect of lumbopelvic malalignment after PAO.

## AUTHOR CONTRIBUTIONS

**Maximilian Fischer**: Conceptualization; methodology; formal analysis; investigation; visualization; writing—original draft preparation; writing—review and editing. **Matthias Mühler**: Conceptualization, methodology; writing—review and editing. **Georgi Wassilew**: Conceptualization; writing—review and editing. **Lars Nonnenmacher**: Formal analysis; investigation; writing—review and editing. **Andreas Nitsch**: Formal analysis; investigation; writing—review and editing. **Alexander Möller**: Formal analysis; investigation; writing—review and editing. **Andre Hofer**: Formal analysis; investigation; writing—review and editing.

## CONFLICT OF INTEREST STATEMENT

Georgi Wassilew has received research support from Enovis and Smith & Nephew without relevance to the content of this article. The remaining authors declare no conflicts of interest.

## ETHICS STATEMENT

Institutional review board approval (BB099/20a) was obtained from the local independent ethics committee of the University Medicine Greifswald according to the World Medical Association Declaration of Helsinki. All patients gave written informed consent prior to inclusion.

## Data Availability

The data that support the findings of this study are available from the corresponding author upon reasonable request.
